# Membrane Properties and the Balance between Excitation and Inhibition Control Gamma-Frequency Oscillations Arising from Feedback Inhibition

**DOI:** 10.1371/journal.pcbi.1002354

**Published:** 2012-01-19

**Authors:** Michael N. Economo, John A. White

**Affiliations:** 1 Department of Biomedical Engineering, Boston University, Boston, Massachusetts, United States of America; 2 Department of Bioengineering, Brain Institute, University of Utah, Salt Lake City, Utah, United States of America; University of Freiburg, Germany

## Abstract

Computational studies as well as *in vivo* and *in vitro* results have shown that many cortical neurons fire in a highly irregular manner and at low average firing rates. These patterns seem to persist even when highly rhythmic signals are recorded by local field potential electrodes or other methods that quantify the summed behavior of a local population. Models of the 30–80 Hz gamma rhythm in which network oscillations arise through ‘stochastic synchrony’ capture the variability observed in the spike output of single cells while preserving network-level organization. We extend upon these results by constructing model networks constrained by experimental measurements and using them to probe the effect of biophysical parameters on network-level activity. We find in simulations that gamma-frequency oscillations are enabled by a high level of incoherent synaptic conductance input, similar to the barrage of noisy synaptic input that cortical neurons have been shown to receive *in vivo*. This incoherent synaptic input increases the emergent network frequency by shortening the time scale of the membrane in excitatory neurons and by reducing the temporal separation between excitation and inhibition due to decreased spike latency in inhibitory neurons. These mechanisms are demonstrated in simulations and *in vitro* current-clamp and dynamic-clamp experiments. Simulation results further indicate that the membrane potential noise amplitude has a large impact on network frequency and that the balance between excitatory and inhibitory currents controls network stability and sensitivity to external inputs.

## Author Summary

The gamma rhythm is a prominent, 30–80-Hz EEG signal that is associated with cognition. Several classes of computational models have been posited to explain the gamma rhythm mechanistically. We study a particular class in which the gamma rhythm arises from delayed negative feedback. Our study is unique in that we calibrate the model from direct measurements. We also test the model's most critical predictions directly in experiments that take advantage of cutting-edge computer technologies able to simulate ion channels in real time. Our major findings are that a large amount of “background” synaptic input to neurons is necessary to promote the gamma rhythm; that inhibitory neurons are specially tuned to keep the gamma rhythm stable; that noise has a strong effect on network frequency; and that incoming sensory input can be represented with sensitivity that depends on the strength of excitatory-excitatory synapses and the number of neurons receiving the input. Overall, our results support the hypothesis that the gamma rhythm reflects the presence of delayed feedback that controls overall cortical activity on a cycle-by-cycle basis. Furthermore, its frequency range mainly reflects the timescale of synaptic inhibition, the degree of background activity, and noise levels in the network.

## Introduction

The 30–80 Hz gamma rhythm is among the most prominent and ubiquitous forms of rhythmic activity in the brain [1]–[5]. Since its discovery, a multitude of functions and mechanisms have been ascribed to this form of oscillatory behavior. The gamma rhythm has been proposed as a requirement for sensory binding, attention, and memory formation [6]–[10], and disruption of the rhythm has been suggested as a critical factor in pathologies such as schizophrenia, autism, and epilepsy [11]–[15]. The gamma rhythm has been modeled extensively and multiple mechanistic hypotheses have been introduced to explain its origin [15]–[17].

Mechanistically, the gamma rhythm was initially studied experimentally in networks of pharmacologically-isolated interneurons [18]. These experiments motivated theoretical studies that sought to connect previous results concerning synchronization mediated by inhibition [19] with experimental observations on the gamma rhythm [18], [20]–[24]. These studies were successful in devising a mechanistic explanation of synchronization among interneurons and helped explain several key experimental findings, such as the observation that the frequency of network oscillations is closely related to the decay kinetics of inhibition and the conditions under which the oscillation may be stabilized in the presence of heterogeneity. While these and other findings seem to be generalizable to gamma rhythms arising by other mechanisms, it has become clear that this rhythm depends on other cell populations in addition to interneurons under many experimental conditions [16], [17].

A synchronization mechanism solely involving interneurons may straightforwardly be adapted to networks including excitatory neurons if one assumes that the excitatory neurons are simply entrained by inhibition from an oscillating interneuronal population. However, experimental studies have established that gamma rhythms may be generated by inducing activity in excitatory cells [25], that the gamma period is correlated with excitatory neuron activity levels [26], and that gamma rhythms persist in the absence of GABAergic transmission between interneurons [27]. While interneurons are still thought to play a vital role in gamma rhythmogenesis, these findings imply that principal neurons are not simply entrained by an oscillating interneuron population.

A separate series of studies of the gamma rhythm and other rhythms *in vivo* and *in vitro* indicate that both excitatory and inhibitory cells fire sparsely and irregularly, even when robust oscillations are recorded at the population level [28]–[34]. Therefore, it is likely that gamma rhythmogenesis might be difficult to explain through the study of synchronization in neurons oscillating in a periodic manner. An attractive alternative hypothesis is that the gamma rhythm arises as a consequence of coherent feedback inhibition recruited by irregularly-firing excitatory neurons. This ‘stochastic synchrony’ has been studied in reduced, idealized networks [35], [36] and in large-scale detailed models [37], [38] and can possess fundamentally different dynamical structures at the network level [39].

In this study, we use experimental measurements of the biophysical properties of excitatory and inhibitory neurons to constrain simulated networks generating a gamma rhythm through a stochastic feedback mechanism. Our simulations suggest that the period of the gamma rhythm and its stability are strongly dependent upon the balance and variability of excitatory and inhibitory synaptic conductances impinging upon neurons in the local network. We provide an explanation of these dependencies and we present evidence that the stability and sensitivity of the network to external input is controlled by the balance of excitation and inhibition in magnitude and in time.

## Materials and Methods

### Ethics statement

All experimental protocols were approved by the University of Utah Institutional Animal Care and Use Committee.

### Tissue preparation

Coronal sections of neocortex were prepared from 15- to 28-day old G42 mice in which GABAergic, parvalbumin-positive interneurons are fluorescently labeled with GFP. G42 transgenic mice [40] were obtained from The Jackson Laboratory (http://www.jax.org). All chemicals were obtained from Sigma-Aldrich unless otherwise noted. After the animals were anesthetized with isoflurane (VETone) and decapitated, brains were removed and immersed in 0°C artificial CSF (aCSF) consisting of the following (in mM): 125 NaCl, 25 NaHCO_3_, 25 D-glucose, 2.5 KCl, 2 CaCl_2_, 1.25 NaH_2_PO_4_, and 1 MgCl_2_ (buffered to pH 7.4 with 95% O_2_/5% CO_2_). Coronal slices were cut to a thickness of 400 µm with a vibrating microtome (VT1200; Leica Microsystems). Slices were then incubated at room temperature (∼22°C) in oxygenated aCSF for 60 min. After the incubation period, slices were moved to the stage of a custom-built microscope equipped with brightfield and two-photon fluorescence optics. Slices were bathed in standard aCSF with 10 µM CNQX and 50 µM picrotoxin added in some experiments to block synaptic activity. All recordings were conducted at 33°C.

### Electrophysiology

Electrodes were drawn on a horizontal puller (P97; Sutter Instruments) and filled with an intracellular solution consisting of the following (in mM): 120 K-gluconate, 20 KCl, 10 HEPES, 7 diTrisPhCr, 4 Na_2_ATP, 2 MgCl_2_, 0.3 Tris-GTP, 0.2 EGTA and 0.6% biocytin (Invitrogen) by weight, and buffered to pH 7.3 with KOH. Final electrode resistances were between 3 and 4 MOhms, with series access resistance values between 4 and 15 MOhms. Electrophysiological recordings were performed with a current-clamp amplifier (Multiclamp 700A; Axon Instruments), and data were acquired using RTXI (www.rtxi.org).

**Figure 1:**
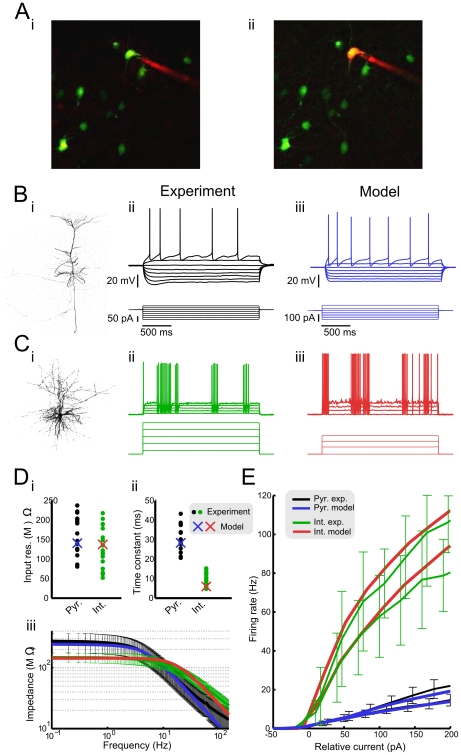
Model neurons were constructed to match electrophysiological data. **A** Fast-spiking interneurons in layer II/III of primary somatosensory cortex were patched in G42 mice that express green fluorescent protein (GFP) in a subpopulation of parvalbumin-positive interneurons. A GFP+ neuron before (*i*) and after (*ii*) a recording in the whole-cell configuration was initiated with a long wavelength fluorophore included in the intracellular solution. **B** Morphology (*i*) and response to constant current steps (*ii*) of a recorded layer II/III regular-spiking pyramidal (RSP) neuron. (iii) Response to constant current steps of model regular-spiking pyramidal neuron. **C** Morphology (*i*) and response to constant current steps (*ii*) of a recorded fast-spiking (FS) interneuron. (iii) Response to constant current steps of model fast-spiking interneuron. **D** Input resistance (*i*), time constant (*ii*), and subthreshold impedance spectra (*iii*) of fast-spiking interneurons and regular-spiking pyramidal neurons from *in vitro* experiments (black, green) and model simulations (blue, red). **E** Firing frequency-current relationships of RSP neurons (experiment, black; model, blue) and FS interneurons (experiment, green; model, red) in the non-adapted (median of first five interspike intervals; upper line) and fully-adapted (median of last five interspike intervals; lower line) conditions.

Fast-spiking interneurons were patch-clamped under fluorescent guidance with a custom-built two photon microscope (Fig. 1A) while pyramidal neurons were selected based on their location in layer II/III, pyramidal-shaped somata, and the presence of a large apical dendrite projecting towards layer I. The identity of all cells was confirmed with post-hoc morphological analysis. All (28/28) recorded cells that expressed GFP displayed a fast-spiking electrophysiological phenotype and were found to have morphology consistent with basket interneurons.

### Morphological reconstructions

All intracellular recordings were performed with intracellular fluid containing 0.6% biocytin by weight. After cells were allowed to passively fill with biocytin for one hour, they were fixed in 4% paraformaldehyde. To visualize neurons, slices were washed three times for 15 minutes in 0.1 M Phosphate buffered saline (PBS) and then incubated for three hours in a solution containing 1 mg/mL streptavidin-Alexa 488 or 532 (Invitrogen) and 0.75 mL/100 mL Triton X-100 (Sigma) in PBS. After another three washes for 15 minutes each in PBS, slices were mounted on microscope slides in Mowiol. Slides were imaged on a custom built 2-photon microscope. Images stacks were processed with ImageJ (NIH) and processes were traced with the Neuromantic tracing software (http://www.reading.ac.uk/neuromantic/).

### Protocols

For dynamic-clamp experiments, the current-clamp amplifier was driven by an analog signal from an ×86 personal computer running Real-Time Application Interface Linux and an updated version of the Real-Time Linux Dynamic Clamp [41] called Real-Time eXperimental Interface [RTXI]; [ 42,43]. The sample rate of the dynamic clamp system was set to 10 kHz. Data were low-pass filtered with a cutoff frequency of 4 kHz and collected at 10 kHz. All stimulation protocols were created using custom plug-in extensions of RTXI. Frequency-current relationships, input resistance, and membrane time constants were determined using a sequence of square current steps lasting 2 seconds with an inter-step interval of 6 seconds. Membrane time constants and input resistances were measured in each cell using subthreshold current steps. To measure membrane impedance spectra, 10 second-long noisy current stimuli were applied to each cell with five repetitions at a constant holding potential of −60 mV. These stimuli were constructed in the frequency domain with a flat frequency spectrum between zero and 200 Hz and random phases. The amplitudes of these stimuli were adjusted to produce a 5 mV peak-to-peak fluctuation in membrane potential to minimize membrane nonlinearities. In experiments measuring membrane responses to simulated inhibitory post-synaptic conductances, bi-exponential conductance waveforms (as in Equation 20) with a rise time constant of 1 ms and reversal potential of −75 mV were applied every 500 ms to a quiescent cell, either simulated (low-conductance state) or biological, while varying the decay time constant, amplitude, and tonic background conductance in random order. Simulated inhibitory conductances with each combination of parameters were applied 6 times to each cell. In order to measure spike latencies, spike threshold was first determined and then each cell was held approximately 10 mV below spike threshold with applied DC current. Simulated post-synaptic conductances with a 1 ms rise time constant, 5 ms decay time constant, and a reversal potential of 0 mV were applied every two seconds with varying amplitude. The ‘threshold’ conductance, *G_th_*, was determined as the minimum amplitude needed to elicit a spike and waveform amplitudes were varied over the range of 0.5*G_th_* to 3.0*G_th_* in steps no larger than 0.15*G_th_*. All amplitudes were presented 6 times in randomized order.

### Model cells

Simulated regular-spiking pyramidal neurons were modeled using a version of the adaptive exponential integrate-and-fire model [44] modified to include an extra time scale of spike-rate adaptation. The current balance equation for the model was:
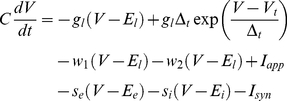
(1)Equation 1 is similar to the leaky integrate-and-fire model with several extra terms. The exponential term gives rise to action potentials with sharpness determined by *Δ_t_*. The variable *w_1_* represents spike-dependent and spike-independent adaptation on a fast time scale. This process prevents the first several ISIs from becoming two short in response to large step currents and prevents unrealistically-high instantaneous firing frequencies in response to strong inputs. In contrast, *w_2_* represents purely spike-dependent adaptation at longer time scales. This slow adaptation reproduces the gradual lengthening in ISIs observed in layer II/III pyramidal neurons in response to the first several hundred milliseconds of a step input (Fig. 1B *ii*). The adapted and unadapted firing frequency-current relationships measured in layer II/III pyramidal neurons (Fig. 1E) could not be reproduced without including two time scales of adaptation in the model. The terms *w_1_* and *w_2_* were determined by the differential equations:
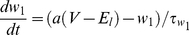
(2)

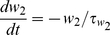
(3)When voltage exceeded the reset threshold of +20 mV, the state variables were modified in the following manner:
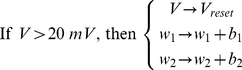
(4)The variables *s_e_* and *s_i_* in Equation 1 represent random conductance fluctuations which were modeled as Ornstein-Uhlenbeck processes [45], [46] and evolved according to the update rule:
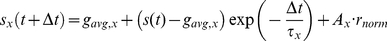
(5)

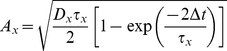
(6)where *r_norm_* was a normally-distributed random variable with zero mean and unitary standard deviation. *g_avg,x_* represents the mean conductance of a process, x, 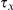 represents the time scale of decay of fluctuations in this conductance, and *D_x_* represents the magnitude of fluctuations in the conductance.

Synaptic current, *I_syn_*, was calculated according to the equation:
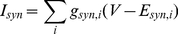
(7)Where *i* enumerates all synapses impinging upon the neuron, *E_syn,i_* is the reversal potential of synapse *i*, and *g_syn,i_*, given by Equation 20, is the conductance of the synapse.

Parameters of the model are given in Table 1.

**Table 1:** Synapse and model parameters.

RSP neuron model	Value	Units	FS interneuron model	Value	Units
*C*	0.15	nF	*C*	1.0	µF/cm^2^
*g_l_*	4.5	nS	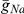	135	mS/cm^2^
*E_l_*	−65	mV	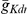	675	mS/cm^2^
*Δt*	0.8	mV	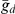	0.3	mS/cm^2^
*V_reset_*	−53	mV	*g_l_*	7.0	nS
*V_t_*	−52	mV	*E_Na_*	50	mV
*a*	−0.00005	nS/mV	*E_K_*	−70	mV
*τ_w1_*	20	ms	*E_l_*	−65	mV
*b_1_*	0.05	nS	*θ_m_*	−24	mV
*τ_w2_*	550	ms	*σ_m_*	11.5	mV
*b_2_*	0.0015	nS	*θ_h_*	−58.3	mV
			*σ_h_*	−6.7	mV
*g_avg,e-low_*	5×10^−4^	nS	*θ_th_*	−60	mV
*g_avg,i-low_*	2×10^−3^	nS	*σ_th_*	−12	mV
*g_avg,e-high_*	10.8	nS	*θ_n_*	−1.24	mV
*g_avg,i-high_*	2.7	nS	*σ_n_*	−9.8	mV
*D_e_*	5×10^−4^	nS^2^/ms	*θ_a_*	−50	mV
*D_i_*	2×10^−3^	nS^2^/ms	*σ_a_*	20	mV
*τ_e_*	2	ms	*θ_b_*	−70	mV
*τ_i_*	8	ms	*σ_b_*	6	mV
*E_e_*	0	mV	*τ_w_*	500	ms
*E_i_*	−75	mV			
			*g_avg,e-low_*	5×10^−4^	nS
**Synapse parameters**	**Value**	**Units**	*g_avg,i-low_*	2×10^−3^	nS
*τ_rise,E_*	0.5	ms	*g_avg,e-high_*	12.0	nS
*τ_rise,I_*	1.0	ms	*g_avg,i-high_*	3.0	nS
*τ_fall,E_*	2.5	ms	*D_e_*	5×10^−4^	nS^2^/ms
*τ_fall,I_*	5.0	ms	*D_i_*	2×10^−3^	nS^2^/ms
*E_syn,EE_*	0	mV	*τ_e_*	2	ms
*E_syn,EI_*	0	mV	*τ_i_*	8	ms
*E_syn,IE_*	−65	mV	*E_e_*	0	mV
*E_syn,II_*	−55	mV	*E_i_*	−75	mV

Cortical fast-spiking interneurons were modeled using a published conductance-based model [47] with modifications to fit our experimental measurements. The current-balance equation for the model was:
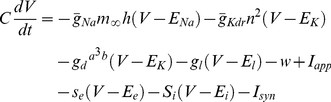
(8)The model included a fast, inactivating sodium conductance, a delayed rectifier potassium conductance, a D-type potassium conductance, and a leak conductance, each of which were modeled according to the formalism of Hodgkin and Huxley [48]. Additionally, the model included a simple adaptation variable w, which evolved in analogous fashion to the variable w_2_ described in Equation 3. The variables *s_e_* and *s_i_* were as given in Equations 5 and 6. The equations governing the transient sodium conductance were as follows:
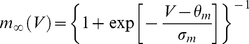
(9)

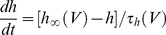
(10)

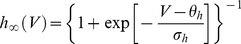
(11)

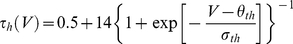
(12)where the activation variable, *m*, was an instantaneous function of voltage. The gating variables of the delayed rectifier potassium conductance evolved according to:
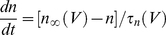
(13)

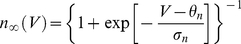
(14)

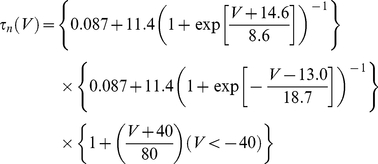
(15)The model also included a D-type potassium conductance. It was described by:
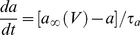
(16)

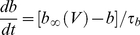
(17)

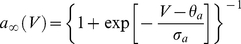
(18)

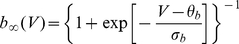
(19)
*τ_a_* and *τ_b_* were 2 ms and 150 ms respectively. Again, all model parameters are given in Table 1. Model interneurons were represented as isopotential spheres with a radius of 18 µm so that input resistance, capacitance, and applied current values in non-normalized units could be compared to the analogous experimentally-relevant quantities. The value of 18 µm was chosen so that model cells and experimentally-recorded interneurons had similar input resistance and time constant values.

### Network architecture

Simulations consisted of two populations of 400 excitatory regular-spiking pyramidal (RSP) neurons and 100 inhibitory fast-spiking (FS) interneurons. Connection probabilities, *p_ij_*, between a presynaptic neuron *i* and a postsynaptic neuron *j* were determined by the identity of pre- and post-synaptic cells according to *p_EE_ = 0.2, p_II_ = 0.4, p_EI_ = 0.4*, and *p_IE_ = 0.4* where E represents an excitatory cell and I represents an inhibitory cell. The connectivity was sparser between excitatory neurons than between other pairs [49]. The effect of synaptic transmission was modeled by the initiation of a bi-exponential conductance waveform in a postsynaptic cell immediately following a zero-crossing of membrane voltage in any presynaptically-connected cell according to:
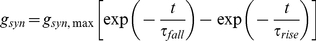
(20)A further normalization constant was employed in simulations in which synaptic decay time constants were altered. This constant was equal to 
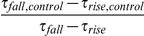
, and ensured that the total time-integrated conductance initiated in the postsynaptic cell remained constant in simulations testing the contribution of synaptic kinetics on network period. To determine synaptic conductance magnitudes, the total postsynaptic conductance from all cells of a given type (i.e. excitatory) onto an (excitatory) cell *j*, 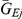, was first sampled from a uniform distribution with minimum 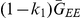 and maximum 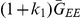. Individual synapse magnitudes 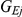 were then chosen from a uniform distribution with minimum 
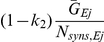
 and maximum 
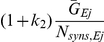
, where 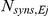 would be the total number of excitatory synapses onto cell *j*. Synapses connecting other populations were determined in an analogous fashion. In this way, the variability in the total synaptic conductance impinging upon each cell and the variability in individual synaptic conductances could be independently controlled by the parameters *k_1_* and *k_2_* respectively. In additional simulations conducted to test the effects of *k_1_* and *k_2_* on network activity no strong dependence was observed (data not shown), and so we make *k_1_* and *k_2_* identical and equal to 0.25. Total synaptic conductances are given in Table 2. Although the decay time constant of inhibition impinging upon interneurons has been shown to be shorter than for inhibition onto principal cells [50], these values were taken to be identical for simplicity and because network simulations were not found to be sensitive to the time scale of interneuron-interneuron connections (Supporting Fig. S1). Synaptic conductances in high-conductance state simulations were determined empirically so that post-synaptic voltage deflections remained constant.

**Table 2:** Maximal synaptic conductance values used in simulations of the high- and low-conductance states.

Low conductance synapses	Value	Units
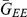	37.5	nS
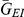	100	nS
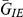	46.25	nS
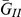	25	nS

### Simulations

**Figure 2:**
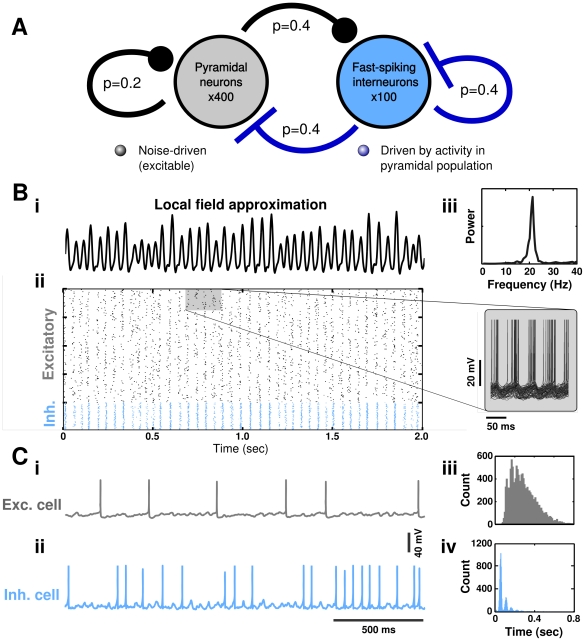
Emergent oscillations arise in a model network due to feedback inhibition. **A** Schematic of network architecture (detailed in *Materials and Methods*). **B** Field potential approximation (*i*) and spike rastergram (*ii*) of cells in the model network. The power spectrum of the field potential approximation (*iii*) clearly indicates an emergent oscillation at the network level at approximately 20 Hz. Inset illustrates loose synchronization in a subset of 75 RSP neurons. **C** Despite a clear oscillation at the population level, such an oscillation is obscured by irregular fluctuations in individual membrane potential traces from RSP neurons (gray) and FS interneurons (light blue). Model pyramidal neurons spike sparsely on approximately every tenth cycle of the oscillation while model interneurons spike every other cycle, on average. Interspike interval histograms of RSP neurons (*iii*) and FS interneurons (*iv*) are highly variable, although an oscillation is clearly visible in the FS interneuron histogram.

**Figure 3:**
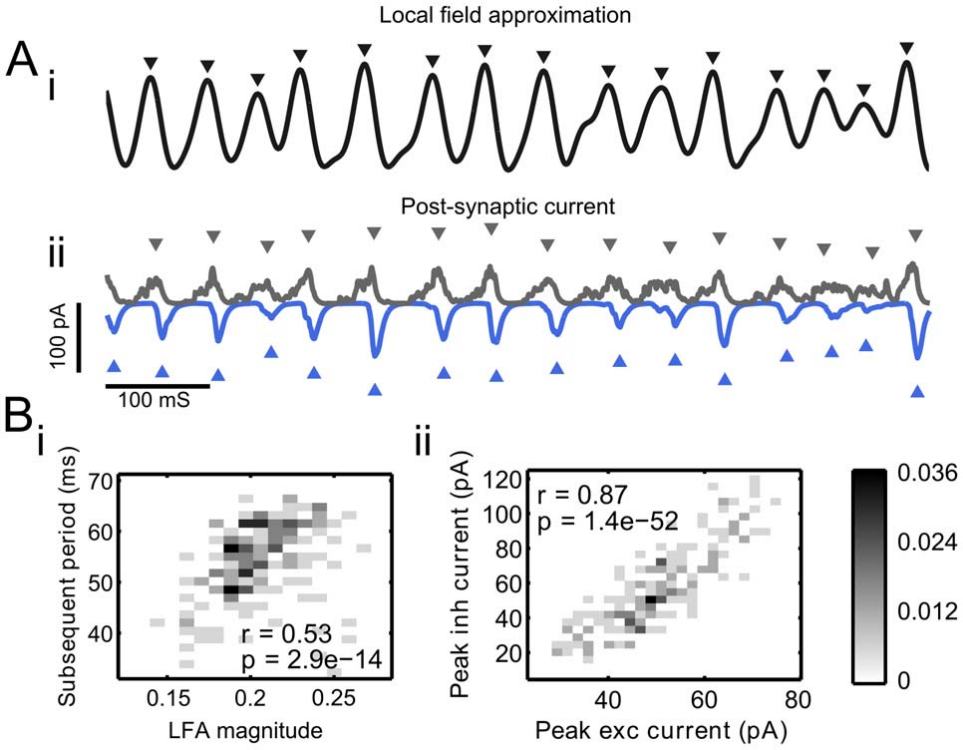
Balance of excitatory and inhibitory currents in the network. **A** Synaptic currents are highly phasic, but variable from cycle to cycle, with inhibition (blue trace) lagging excitation (black trace) by several milliseconds on each cycle of the oscillation. **B** Histogram of the magnitude and subsequent period of each cycle of the field potential approximation (*i*). The magnitude of the field potential approximation on one cycle is predictive of the subsequent network period, as observed in experimental data [26]. (*ii*) Histogram of the amplitude of excitatory and inhibitory currents on a cycle-to-cycle basis. The magnitudes of these currents are highly correlated. Scale bar is identical for both (*i*) and (*ii*) and represents the fraction of cycles falling into each bin.

**Figure 4:**
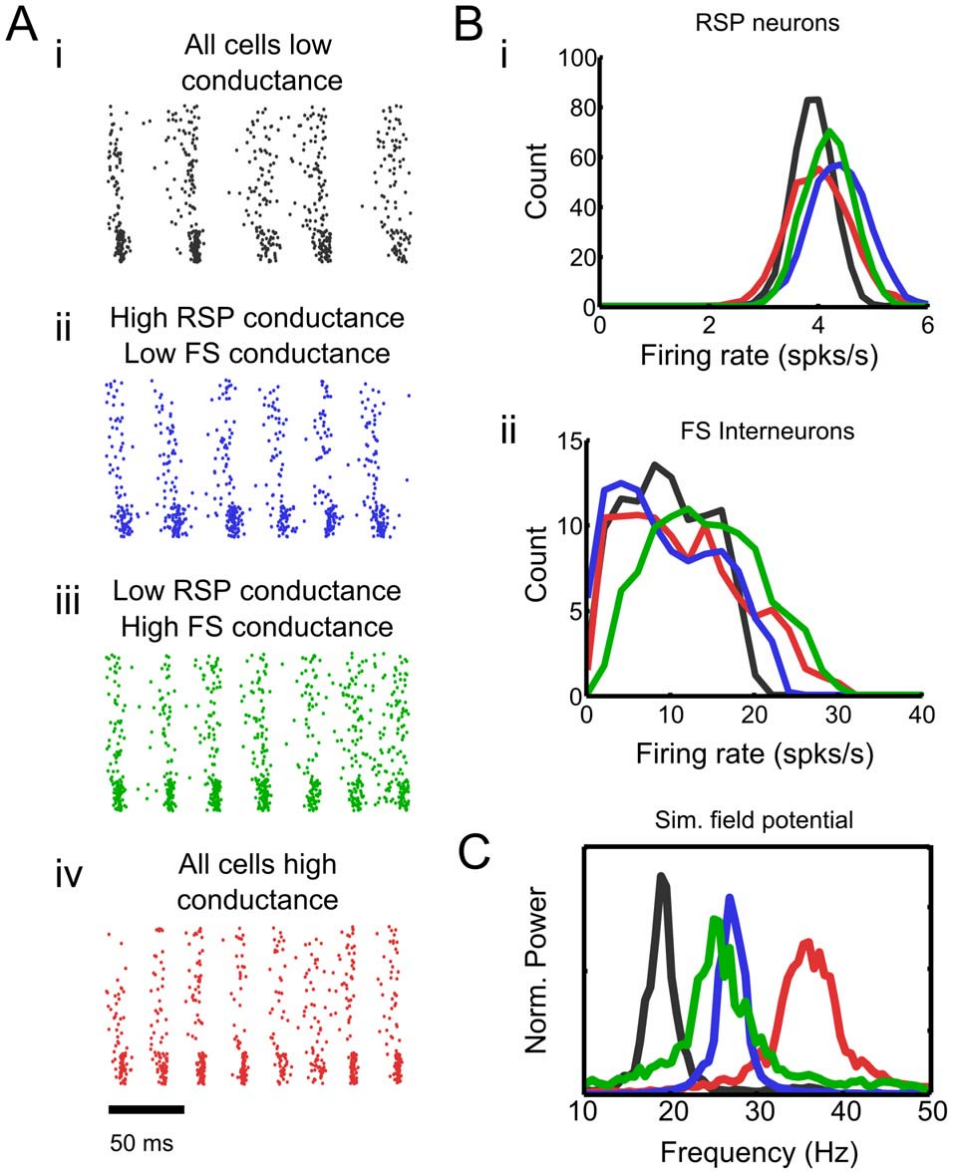
Frequency of network oscillations depends strongly on background conductance in pyramidal neurons and interneurons. **A** Increasing background conductance in pyramidal cells (*ii*; blue) or interneurons (*iii*; green) increases network frequency compared with control (*i*; black). Increasing background conductance in both populations increases network frequency further (*iv*; red). **B** Changes in network frequency arise independent of changes in firing rates of RSP neurons (*i*) or FS interneurons (*ii*). **C** Power spectra of the field potential approximations from the simulations described in *A*.

All simulations were conducted using GenNet [51], a network simulator written in C++. Equations were integrated with a forward Euler solver with a time step of 0.01 ms. Simulation convergence was checked by repeating a subset of simulations with a time step of 0.005 ms and ensuring consistency of solutions. Results in Figs. 2 and 3 were taken from a single ten-second simulation. The simulation data presented in Figs. 4–7 were averages of ten five-second realizations of the model network. Figs. 8 and 9 were generated from 81 two-second simulations and 800 one-second simulations, respectively.

**Figure 5 pcbi-1002354-g005:**
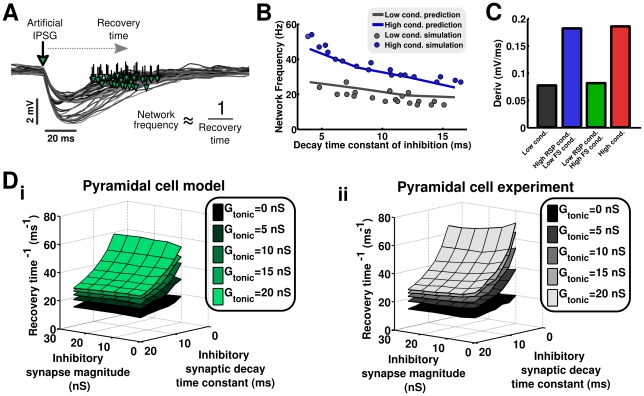
Recovery time after inhibition in pyramidal neurons controls network frequency. **A** Recovery time after inhibition was determined by simulating an inhibitory synaptic conductance waveform in isolated model pyramidal neurons and in quiescent biological neurons *in vitro* via dynamic clamp. The example illustrated was taken from a representative dynamic clamp experiment. These simulated synaptic conductances varied in their magnitude, decay kinetics, and in the amount of tonic background conductance present. **B** Predictions of network frequency (lines), taken as the inverse of measured recovery time after inhibition, match the frequency of network oscillations determined in full simulations (solid dots) when RSP neurons are in either a low conductance state (gray) or high conductance state (blue). **C** The derivative of membrane voltage following inhibition in model pyramidal neurons in the oscillating model network depends upon the conductance state of those cells. RSP neurons in a low-conductance state (gray, green) recover slowly following inhibition, while RSP neurons in a high-conductance state (blue, red) recover relatively quickly. **D** Mean values of the inverse of recovery time after inhibition in model RSP neurons (*i*) and biological layer II/III pyramidal neurons (*ii*) as a function of decay time constant and synapse magnitude (axes) and background conductance (surfaces). Recovery time is controlled by the decay time constant of inhibition and total level of membrane conductance but not the magnitude of phasic conductance in model and biological neurons.

**Figure 6 pcbi-1002354-g006:**
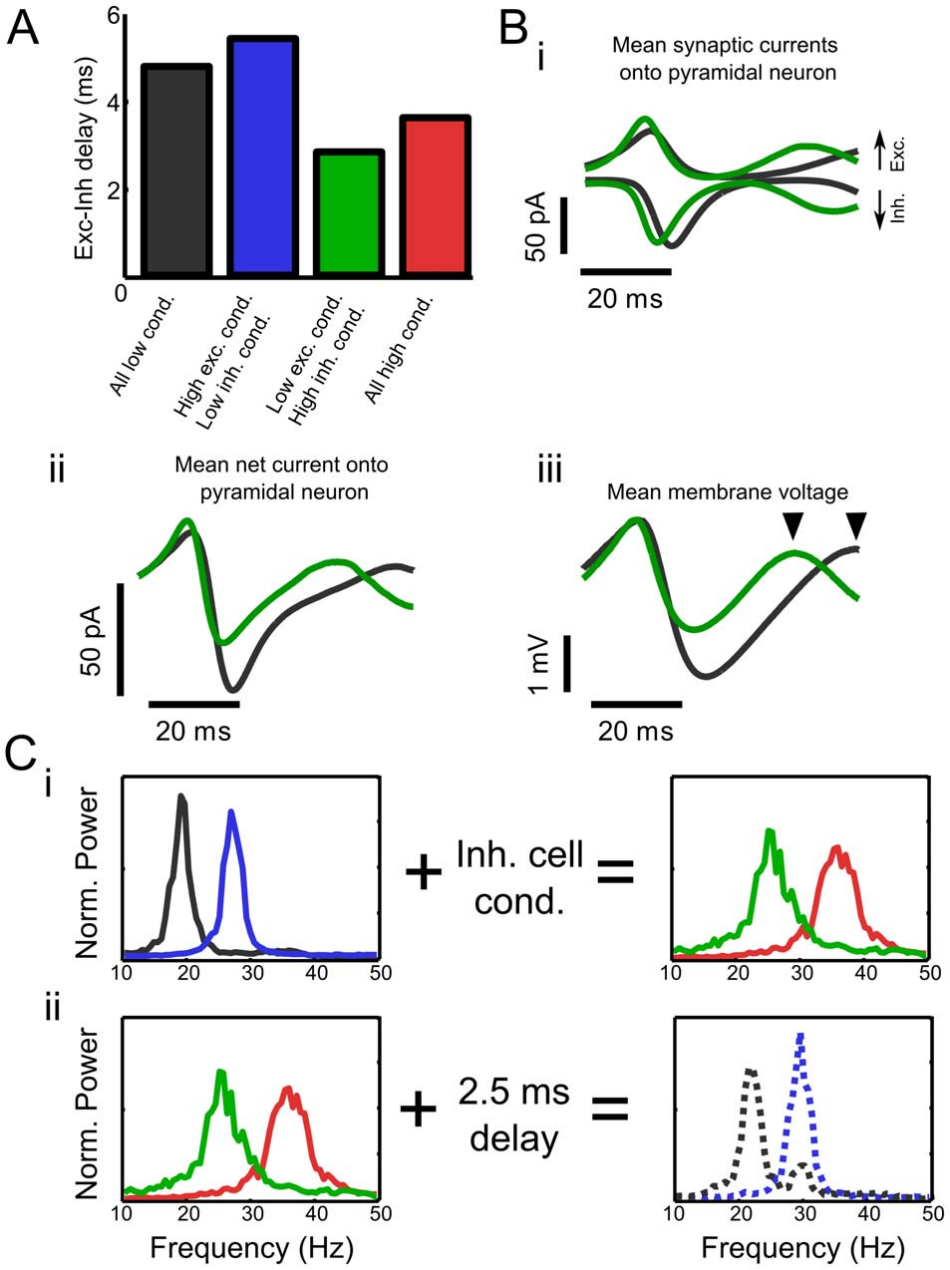
Conductance in interneurons increases network frequency by decreasing the latency with which inhibition is recruited. **A** The delay between peak excitatory and inhibitory currents in pyramidal neurons is shorter in simulations in which background conductance is added to the interneuronal population. **B** Average inhibitory and excitatory current waveforms (*i*) onto a single pyramidal neuron in network simulations with all cells in the low-conductance state (black) and with only interneurons in a high-conductance state (green). The additional peak in the high-conductance state depicts the following network period (clipped for the low-conductance state due to its longer period). Decreasing the latency with which inhibition is recruited balances excitatory and inhibitory currents to a much greater degree (*ii*), resulting in more modest postsynaptic effect of inhibition (*iii*). **C** Adding background conductance to interneurons in the low conductance state increases the frequency of network oscillations (*i*). This effect on network frequency is reversed by the addition of an extra artificial delay in synapses between model interneurons and pyramidal neurons (*ii*). This result indicates that, indeed, the conductance state of interneurons controls the frequency of network oscillations by affecting the temporal balance between excitatory and inhibitory currents in model pyramidal neurons.

**Figure 7:**
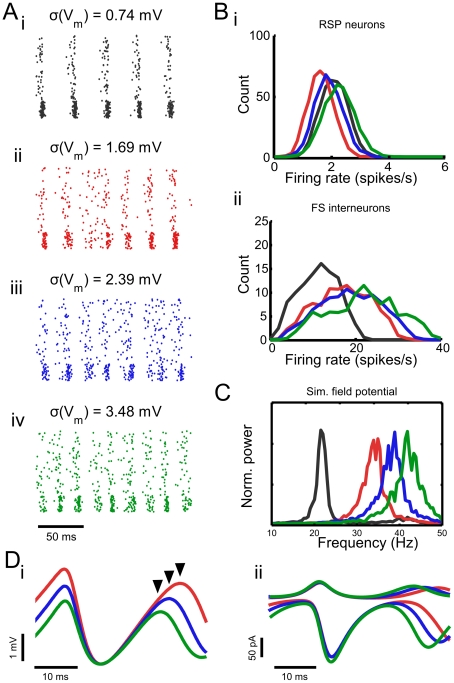
Noise magnitude affects oscillation frequency by shortening recovery time following inhibition. **A** Spike rastergrams of simulations with increasing noise magnitudes (*i–iv*). Indicated standard deviations were determined in the absence of synaptic input. **B** DC currents were adjusted so that the distributions of spike rates in RSP neurons (*i*) remained approximately unchanged across these conditions while spike rates in FS interneurons were distributed between zero and the network frequency (*ii*). **C** Power spectra of the field potential approximations from the simulations shown in *A* indicate an increase in network frequency with increasing noise magnitude. **D** Mean membrane potential of RSP neurons in representative simulations depicted in *A* (*i*) with voltage minima aligned for clarity and mean synaptic currents received by RSP neurons in the same simulations (*ii*).

**Figure 8:**
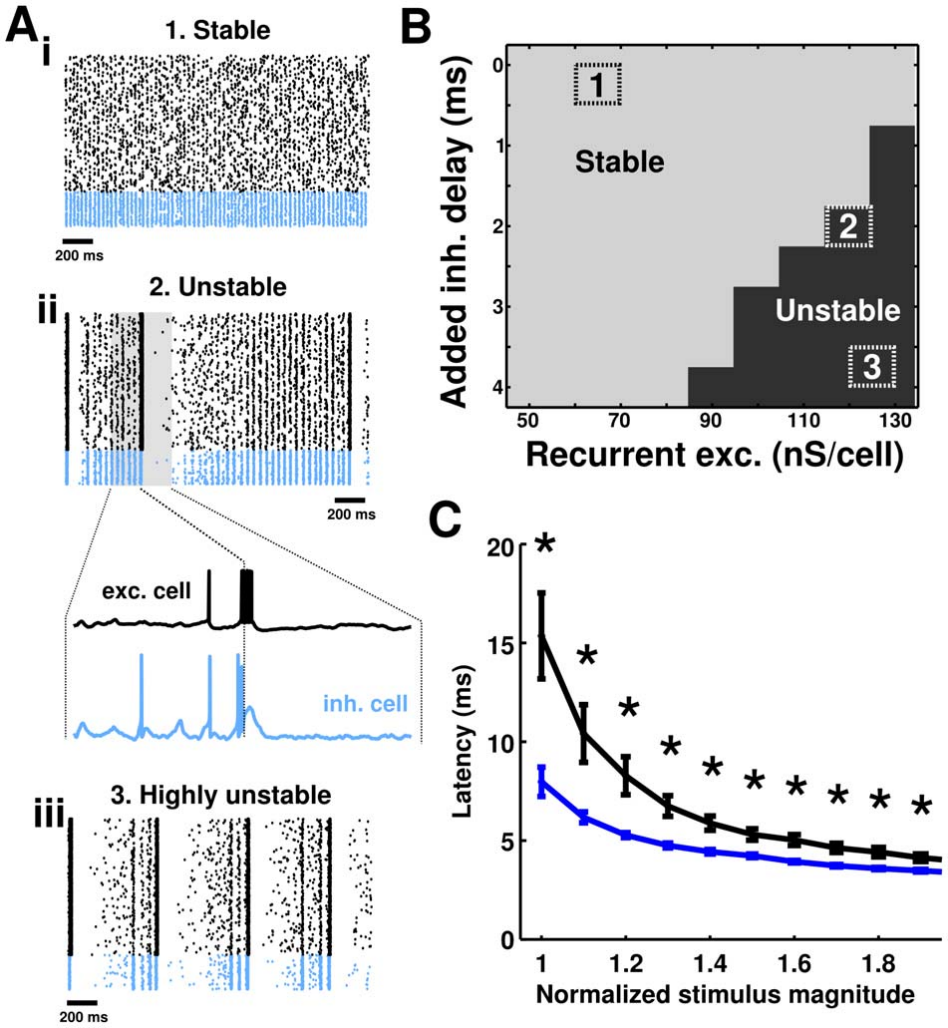
Stability of model oscillation. Unbalancing excitation and inhibition in time or in amplitude transitions the network from stable oscillations to an unstable state in which oscillations are mixed with hypersynchronous bursts of activity. **A** Spike rastergrams of RSP neurons (black) and FS interneurons (blue) from simulations in which excitation and inhibition are approximately balanced (*i*), unbalanced (*ii*), and highly unbalanced (*iii*). **B** Summary of simulations in which synaptic delay and recurrent excitation were varied. Unbalancing excitation and inhibition temporally by adding an extra delay to the inhibitory-to-excitatory cell synapse, or in amplitude by increasing the strength of recurrent inhibitory synapses, produces instability in the network. The parameter combinations for the three simulations in A are depicted in *B*. **C** The response latencies of FS interneurons and RSP neurons *in vitro* to simulated excitatory postsynaptic conductance waveforms introduced via dynamic clamp. The fast-spiking electrophysiological phenotype responds with smaller latency, thereby providing feedback inhibition that effectively balances excitatory currents temporally to maintain stability (n = 8 pyramidal neurons; n = 6 interneurons). *p<0.05 (Wilcoxon rank-sum test).

**Figure 9:**
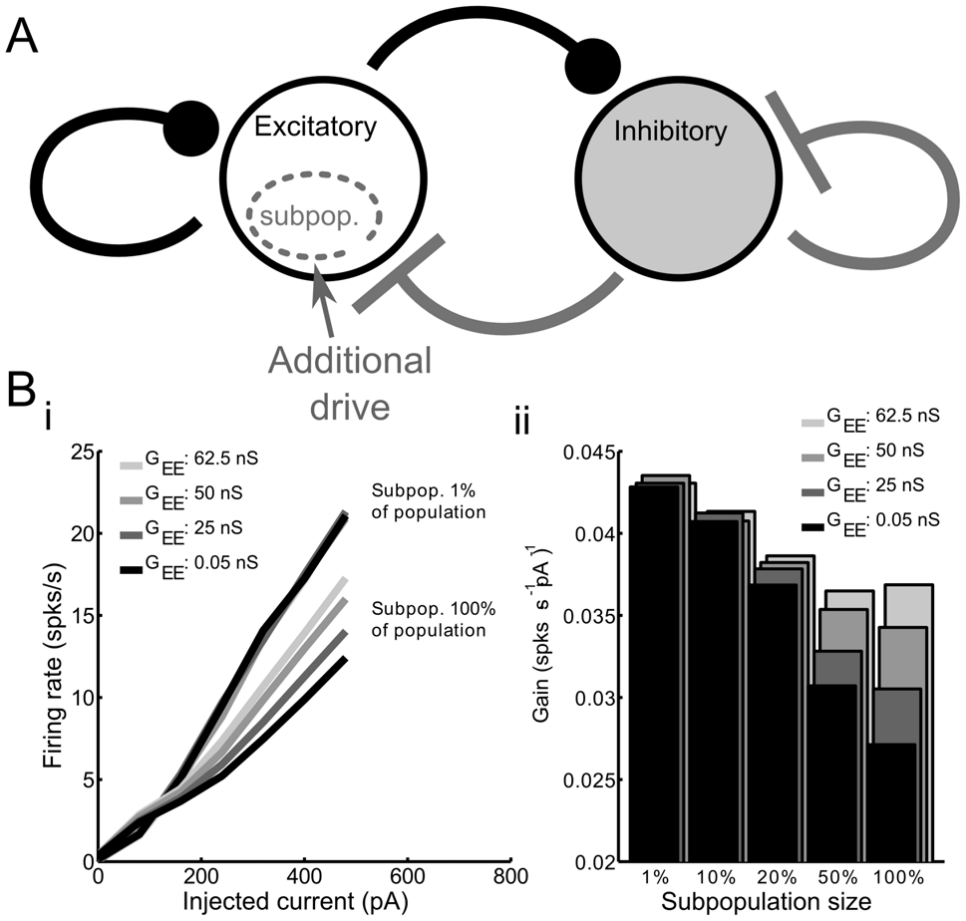
Network sensitivity is determined by the balance between excitation and inhibition. **A** Schematic of manipulation in which additional constant current drive was added to a subpopulation of excitatory cells of variable size. **B** When a small subpopulation (1% of all RSP neurons) receives additional current input, they respond with a relatively large change in firing rate (high gain) (*i*). When the whole network (100%) receives this additional input, proportional recurrent excitation and feedback inhibition are recruited onto each cell, resulting in smaller changes in firing rate (lower gain). The difference in gain between small and large subpopulations depends upon the balance between excitation and inhibition (shifted by manipulating the level of recurrent excitation, represented by the parameter G_EE_). Gain is unaffected by G_EE_ when the subpopulation is small (1%; lines are overlapping). (*ii*) Gain of the firing-frequency current relationship as function of subpopulation size and recurrent excitation. In order to recruit recurrent excitation and feedback inhibition in a manner that affects network sensitivity, the subpopulation must be greater than ∼20% of the cells in the network.

The data described in Supporting Fig. S1 represent average values taken from ten five-second realizations. Parameters and other details regarding these simulations can be found in Supporting Text S1 and Supporting Table S1.

In all simulations comparing the effects of added background conductance on network period, the values of applied current were normalized empirically so that the resting membrane potentials (in the absence of synaptic input) were uniformly distributed between 0 and 15 mV below spike threshold. Additionally, synapse magnitudes were scaled to give equal deviations in the post-synaptic membrane potential (Table 2).

### Analysis

All analyses were performed using custom-written scripts in Matlab (version 2009a; The Mathworks). Input resistances were calculated as the slope of the subthreshold voltage-current relationship produced from a series of subthreshold current steps. Membrane time constants were calculated by fitting an exponential function to the membrane voltage trajectory following the onset of the current steps. Unadapted firing rates were calculated as the median of the inverse of the first five interspike intervals after the onset of a current step. Adapted firing rates were calculated as the median of the inverse of the last five interspike intervals. Impedance spectra were calculated by dividing the magnitude of the fast Fourier transform (FFT) of the membrane voltage by the magnitude of the FFT of the input current. Impedance spectra and firing frequency-current relationships are displayed as mean values with error bars denoting one standard deviation. Power spectra of simulated field potentials were calculated using the Welch method and a sliding two-sec Hamming window with 95% overlap between segments. Power spectra depicting averages across multiple network realizations were calculated by first computing the spectra corresponding to individual simulations and subsequently computing their average value.

Field potential approximations were calculated first by summing the total number of spikes detected on each time step. The dominant network frequency, *f_net_*, was determined as the frequency corresponding to the peak of the Fourier transform of this signal. The field potential approximation was then calculated by low-pass filtering the summated train of spikes with a fourth-order Butterworth filter with a cutoff frequency of *2f_net_*. This filter was applied in the forward and reverse directions in order to preserve phase information. This signal was used as a smooth time-varying estimate of local network activity, similar to a local field potential. However, lacking any geometry in this model, a more accurate local field potential, which arises from the presence of aligned extracellular currents, could not be calculated.

The coefficient of variation of spike times was calculated as the standard deviation of interspike interval times divided by the mean interspike interval. The vector strength of each cell was determined by taking a vector sum of the phases of all spikes in each cell and reporting the magnitude of the resulting vector. Phase was determined relative to the field potential approximation where the preceding peak of the field potential approximation was defined as phase zero and the subsequent peak as phase 2π. Intermediate times were assigned a phase by linearly interpolating between these two values. Correlations in Fig. 3 are Pearson linear correlation coefficients and *p* values are reported as the estimated probabilities that two variables are uncorrelated under the assumption that each variable is normally distributed.

Recovery time after inhibition was determined by measuring the peak negative deflection in membrane voltage (the trough) relative to baseline and then calculating the elapsed time after the trough until the deflection in membrane voltage had decayed by 63.7% (one time constant). Average derivative values (Fig. 5C) were taken between the time points at which the negative deflection in membrane potential had decayed by 10 and 90 percent.

## Results

### Construction of model neurons

For this study, we constructed model neurons in order to simulate the behavior of neocortical layer II/III regular-spiking pyramidal (RSP) neurons and fast-spiking (FS) interneurons. The pyramidal neuron model was based on the adaptive exponential integrate-and-fire model [aEIF; 44] with parameter values for cortical pyramidal neurons (Fig. 1B *ii,iii*) [52]. A single-compartment, conductance-based model was chosen to represent FS interneurons (Fig. 1C *ii,iii*). This model was based on a previously-published model of neocortical fast spiking interneurons [47] and contained inactivating sodium, delayed rectifier potassium, D-type potassium, and linear leak conductances. These models were chosen because their behavior could be modified to well-approximate the firing patterns and intrinsic properties observed in recorded neurons.

In order to ensure that model neurons reproduced the basic biophysics of simulated cell types, whole-cell patch clamp recordings of the cell types of interest were performed from somatosensory cortex of G42 mice [40]. In the neocortex, G42 mice express green fluorescent protein (GFP) in a subset of parvalbumin-positive fast-spiking interneurons with basket morphology (Fig. 1A,C *i*). Model neurons were subsequently modified so that the input resistance, time constant, impedance spectrum, firing frequency-current relationship, and spike-frequency adaptation of each model cell type was consistent with the corresponding quantity in recorded neurons (Fig. 1D,E). A full description of the modified models is given in *Materials and Methods*.

All of the above-mentioned electrophysiological attributes were reproduced closely with the exception of the steep roll-off of impedance at high frequencies (Fig. 1D *iii*) in both cell types. This attribute could not be captured precisely in either model cell, likely due to the fact that both model cells lacked a spatial structure. However, disparities in the frequency response of model cells at high-frequencies (>100 Hz) were deemed to be a minor inconsistency for the purpose of this study. In general, model neurons were found to approximate the behavior of biological neurons very well. With these parameter values, the RSP neuron model was in the ‘integrator’ regime, in which the transition from rest to tonic spiking is described by a saddle-node bifurcation [52]. It has been argued that this dynamical structure best describes biological pyramidal cells in neocortex [53]. Likewise, the transition to spiking in the FS cell model takes place via a subcritical Andronov-Hopf bifurcation as previously suggested [53]. Realistic spiking transitions were included in each model neuron to increase the likelihood that responses to untested stimuli would be similar to the responses of biological neurons [54]–[56].

### Emergent rhythmic activity

In order to investigate the relationship between intrinsic neuronal properties and resultant network oscillations, we simulated a model network consisting of an excitatory RSP neuron population and a population of inhibitory FS interneurons (Fig. 2A; for full network details, see *Materials and Methods*). All simulated neurons were connected randomly and sparsely with connection probabilities determined by the identity of presynaptic and postsynaptic cells. RSP neurons were not active in the absence of noise and were driven by fluctuations of a noisy conductance process. Variability in average spike rates between cells was the result of an additional randomly-distributed DC current. FS interneurons were driven with noisy fluctuations and a randomly-distributed DC current, but resided largely below threshold in the absence of excitatory input from the RSP neuron population. As anticipated, emergent oscillations were observed in the network under these conditions (Fig. 2B). In the oscillatory condition, individual neurons fired irregularly (Fig. 2C; RSP ISI CV 0.44±0.06, FS ISI CV 0.67±0.20) with low rate (RSP rate 3.46±0.33 spks/s, FS rate 9.19±4.78 spks/s) and principal RSP neurons displayed loose synchronization (Fig. 2B *i,ii*; RSP vector strength 0.61±0.094). The standard deviation of membrane potential in RSP neurons was 2.56 mV±0.06, consistent with observations reported *in vivo* during ongoing rhythmic network states [29], [34], [57], [58]. Additionally, these observations were qualitatively similar in the presence of gap junction coupling between FS interneurons (Fig. S1).

Additionally, the network period, determined from the local field approximation (see *Materials and Methods*), varied considerably from cycle to cycle (mean: 51.7, sd: 12.1 ms) and was correlated with the number of cells that were active during the previous cycle (Fig. 3A,B *i*). This correlation has also been observed during ongoing oscillations *in vivo* and *in vitro* and has been proposed as a signature of oscillations arising due to feedback inhibition [26]. Simulated voltage-clamp recordings of cells in the network revealed that excitatory and inhibitory currents in model RSP neurons were tightly correlated in their magnitude (Fig. 3A,B *ii*) and that inhibitory currents lagged by several milliseconds, consistent with experimental observations during gamma [26], [31], [59] and other behavioral states [60]. The overall magnitudes of excitatory and inhibitory currents were also consistent with experimentally-reported values [26], [31], [61].

Under these conditions, the frequency of emergent network oscillations was found to be approximately 20–25 Hz. This result was somewhat surprising as feedback from FS interneurons has been proposed as a mechanism for the generation of the 30–80 Hz gamma rhythm [17], [31], [36], [37], [62], [63]. In fact, varying parameters controlling network size, network connectivity, membrane noise, and DC bias by 50% or more consistently produced network oscillations ranging from 15–35 Hz (data not shown), suggesting that under these conditions, feedback inhibition in sparsely firing populations produces network oscillations at frequencies more closely resembling beta-frequency oscillations than network gamma. For this reason we conclude that oscillations can arise through a feedback mechanism in networks of regular-spiking pyramidal neurons and fast-spiking interneurons in the fluctuation-driven regime, but that the frequency of these oscillations is below the gamma range when experimentally-measured parameters from neurons in a slice preparation are used.

### Background synaptic conductance enables gamma-frequency oscillations

It has been observed in *in-vivo* electrophysiological recordings that neurons in the neocortex receive a constant bombardment of incoherent synaptic activity in the intact brain [64]–[68]. This high background level of excitatory and inhibitory synaptic conductances has been shown to lower the input resistance and time constant of recorded cells by 50–80% [69] compared to the same cell types *in vitro*, or *in vivo* in the presence of local injections of tetrodotoxin [68]. The random inhibitory and excitatory conductance processes already present as noise sources in the model neurons were modified so that the average conductance of these processes was substantially larger (see *g_avg_* values in Table 1), thereby reducing the input resistance and time constant of both RSP and FS neurons in an *in vivo*-like manner (R_in_ of RSP neurons reduced by 63.7%, FS neurons reduced by 66.0%). Following this manipulation, the frequency of model network oscillations increased substantially (Fig. 4 A,C). When both cell types were in a ‘high-conductance state’, varying network size, network connectivity, membrane noise, and DC bias produced network oscillations spanning the range of 20–60 Hz (data not shown), more closely resembling the frequency range of experimentally-recorded gamma oscillations. Furthermore, examining the effect of background conductance on each model cell type individually revealed an increase in network frequency when one of the two cell types was placed in a high-conductance state and an additional increase in frequency in simulations in which both model cell types received increased background conductance (Fig. 4 A,C). The trends in network frequency described here were also present in networks containing electrical synapses between FS interneurons (Supporting Fig. S1).

In order to make these comparisons, mean firing rates of RSP neurons were held approximately constant by the injection of DC current (Fig. 4B) and the amplitude of excitatory and post-synaptic conductances were normalized so as to produce similarly-sized deviations in membrane potential in the presence of increased background synaptic conductance. For this reason, the low- and high-conductance networks cannot be thought of as the same network immediately prior to and following a sudden change in conductance of synaptic origin. Instead, in to order investigate the mechanisms by which higher frequency oscillations arise in the high-conductance state, we have constructed several distinct networks each characterized by realistic firing rates, levels of variability, and post-synaptic potential magnitudes, while varying only the time scale of the neuronal membranes.

Because increasing membrane conductance in each cell type individually increased network frequency independent of the conductance state of the other cell type, we hypothesized that conductance affected network frequency by distinct mechanisms when added to RSP neurons or FS interneurons. We next endeavored to understand these mechanisms in the model network and determine the applicability of these results to biological cells by performing related dynamic clamp experiments.

### Conductance in pyramidal cells controls network frequency by affecting recovery time after inhibition

A series of experimental studies has established an important role for inhibitory synapse kinetics in determining the period of network gamma oscillations. Related theoretical work has suggested a mechanism for this dependence; the interspike interval of cells in the network is dominated by synaptic inhibition (originating either via a feedback mechanism or from an autonomously synchronized inhibitory population; [16], [17]). Following the onset of inhibition, further spiking initiates only when inhibitory currents have sufficiently decayed. This mechanism typically assumes that the decay time constant of inhibition is the longest relevant time constant in the network [70]. However, our experimental measurements of the membrane time constant in RSP neurons in the quiescent slice (Fig. 1D *ii*) indicate that this time scale (28.6±9.1) in pyramidal neurons is longer than the decay time constant of inhibition (4–12 ms) [50], [71]. If the timescale of the membrane is indeed longer than the timescale of synaptic inhibition, then the slow response speed of the membrane should lengthen the period of network oscillations substantially. To reconcile this discrepancy, it has been argued that the membrane time constant of cells embedded in an oscillating network may be shortened by the transient inhibitory conductances necessary for establishing the gamma rhythm, minimizing the impact of the membrane time constant on network period [70].

We wished to better understand the action of transient (i.e. arising from phasic, feedback inhibition) and constant (i.e arising from incoherent background synaptic input) conductance sources on network oscillations in models based on *in vitro* recordings. To accomplish this, we quantitatively tested the manner in which the synaptic decay time constant, the magnitude of phasic inhibitory synaptic conductance, and the magnitude of tonic background conductance each affect the length of time required for the membrane of model RSP neurons to recover after a brief inhibitory synaptic conductance input (Fig. 5A). We simulated the membrane potential of the excitatory model cell after receiving a brief inhibitory synaptic conductance of variable magnitude, decay kinetics and in the presence of variable tonic background conductance. We further defined ‘recovery time’ as the amount of time it took for the resulting transient membrane hyperpolarization to decay by 63.2% of its maximal value (one time constant). We considered recovery time to be a surrogate for network period in this simple simulation.

To test whether this assumption was valid, we compared the predicted network frequency, computed as the inverse of recovery time, with the frequency of oscillations in simulations of the full network (Fig. 5B) while varying the inhibitory synaptic decay kinetics in cases of low- and high-RSP cell conductance (grey and blue, respectively). Predictions of network frequency calculated from recovery time were found to match the network frequency in simulations of the full network extremely well, given the simplicity of this abstraction, across all values of the parameters tested. Thus, we concluded that the inverse of recovery time after inhibition, as described above, could give us important insights into how other pertinent parameters affect the frequency of emergent network oscillations.

Consistent with previous experimental results [18], [20], the decay time constant of inhibition had a considerable effect on the simulated recovery time after inhibition (Fig. 5B,D *i*). Likewise, the amount of tonic background conductance, representing the conductance change originating from a barrage of synaptic input incoherent with the ongoing oscillation, was found to have a dramatic effect on recovery time (Fig. 5D *i*, *surfaces*). In contrast, we found that the brief inhibitory conductances themselves were unable to meaningfully affect the membrane's recovery time following hyperpolarization. If this were the case, and the feedback inhibition arriving in a phasic manner and necessarily present during a gamma rhythm were capable of producing an effective high-conductance state, as suggested [72], [73], then increasing the magnitude of the brief, phasic inhibitory input would be expected to not only increase the magnitude of the resulting hyperpolarization, but also quicken the recovery from this inhibition as well. However, we have found that this scenario is not possible under the conditions we describe.

These measurements were repeated in *in-vitro* experiments using dynamic clamp to present simulated transient inhibitory synaptic and tonic background conductances to layer II/III pyramidal cells (Fig. 5D *ii*). Results from these experiments closely matched simulations. This finding indicated that there were no subthreshold ionic currents active in recorded layer II/III pyramidal neurons, but unaccounted for in our model, that might qualitatively affect measurements of recovery time. In the full network, examining the voltage trajectory following synaptic inhibition-induced hyperpolarization of RSP neurons, we found that the average derivative of membrane voltage during the recovery phase following inhibition was substantially increased under conditions of added background conductance (Fig. 5C). This finding is consistent with our interpretation that high tonic conductance in RSP neurons results in faster recovery of the membrane following inhibitory synaptic input. For this comparison, we used the average derivative as a measurement of recovery speed rather than recovery time after inhibition as recovery time could not be calculated in an accurate manner during population oscillations. We therefore conclude that the recovery time is a very good predictor of network (but not cellular) frequency in stochastically synchronous, fluctuation-driven networks. Furthermore, we conclude that both the time constant of the RSP neuron membrane and the decay kinetics of inhibition have a substantial impact on network frequency, and that gamma frequency oscillations emerge in such networks only in the *in vivo*-like high-conductance state.

### Conductance in inhibitory cells controls network frequency by affecting spike latency

As activity in the FS interneuron population was not limited by inhibition, but rather driven by excitation, we hypothesized that changing the rate at which inhibitory cells recover from inhibition could not have a large effect on network frequency. This hypothesis was supported by simulation results, which showed that the kinetics of inhibitory synapses impinging on inhibitory cells did not significantly affect network frequency (data not shown). For this reason, we concluded that a different mechanism must be responsible for the increase in network frequency observed when background synaptic conductance was added to model FS interneurons.

One striking difference between network simulations containing low- or high-conductance interneuron models was found to be the degree of temporal balance between excitation and inhibition. More specifically, the delay between peak excitatory current and peak inhibitory current in ‘voltage-clamped’ model RSP neurons decreased by approximately 40% when an *in vivo*-like level of background conductance was added to the interneuron population (Fig. 6A). While this reduction in the delay between excitatory and inhibitory currents was modest compared to the network period, the temporal balance between excitation and inhibition was found to be greatly enhanced under this condition (Fig. 6B *i*) leading to a dramatically altered average postsynaptic current waveform in the pyramidal cell population (Fig. 6B *ii*). The total time-integrated net current, calculated over the portion of the oscillation cycle in which current was net inhibitory, was reduced from −0.385 nA-ms in the low conductance condition to −0.1456 nA-ms in the case of high-conductance interneurons - a 62% reduction.

Whereas increasing background conductance in the pyramidal cell population altered the time scale of membrane recovery after inhibition, we hypothesized that increasing background conductance in interneurons decreased their latency to spiking after receiving excitatory synaptic input. A decrease in spike latency would thereby balance the excitation and inhibition received by excitatory neurons more precisely in time (Fig. 6B *i*), and as a result, reduce the amount of net current perceived by postsynaptic RSP neurons (Fig. 6B *ii*). This hypothesis was easily tested in the model network by adding an additional, artificial, synaptic delay into the synapse model to imitate the effect of increased spike latency in model interneurons. Fig. 6C illustrates that adding background conductance to the interneuron population increases the resultant network frequency regardless of the conductance state of the pyramidal cell population (Fig. 6C *i*). Inclusion of a subsequent synaptic delay lowers network frequency to values similar to that observed in ‘low-conductance state’ interneuron simulations (Fig. 6C *ii*). Moreover, examining cycle-averaged membrane potential trajectories in the simulations supports the interpretation that temporally-balanced excitation and inhibition lead to shorter network periods as inhibition is less effective at hyperpolarizing the post-synaptic cell (Fig. 6B *iii*). Examining the cycle-averaged membrane potential trajectories (Fig. 6B *iii*) also illustrates that RSP neurons recover from inhibition at the same rate, regardless of the conductance state of the FS interneuron population. Although adding background conductance to RSP neurons also reduces spike latency, this alteration has very little effect on network period as it does not change the temporal balance between excitatory and inhibitory currents in those cells.

### Membrane noise controls network frequency by affecting recovery time after inhibition

Interestingly, we found that network frequency was sensitive to the magnitude of membrane noise as well as membrane conductance. For all parameter combinations, increasing the firing rate of individual neurons increased network frequency. However, when the membrane noise magnitude was increased in the population of model RSP neurons (by manipulating parameters *D_e_* and *D_i_*) and the commensurate increases in firing rate were compensated for with a decrease in mean DC current, increases in network frequency were found to persist (Fig. 7A–C). Although this manipulation alters the noisy conductance processes controlling the conductance ‘state’ of model neurons, fluctuation magnitudes remained small compared to the mean conductances of these processes. For this reason, it was possible to control the magnitude of membrane fluctuations without impacting membrane time constant. The effect of membrane noise on oscillation frequency can be easily understood in the context of recovery time following inhibition. The quiescent portion of an oscillation cycle, when inhibition is maximal, ends when the most depolarized cells in the network are able to spike. Empirically, we found that this condition was satisfied in the control network when inhibition-induced membrane hyperpolarization had decayed by approximately 63.7% (one time constant). In essence, increasing the level of membrane noise produces threshold crossings in the most depolarized neurons earlier in the cycle. This effect can be seen in Fig. 7D, which illustrates that cycles of the oscillation end earlier when noise is increased, at a point in time when most of the cells remain increasingly hyperpolarized (Fig. 7D *i*; arrowheads). Cycles of the oscillation are terminated at an earlier point in time even though the magnitudes of the synaptic currents received in each condition are nearly indistinguishable across the simulations (Fig. 7D *ii*). This result strongly supports our suggestion that recovery time following inhibition is a good predictor of network frequency. Furthermore, the results from Fig. 7 imply that the magnitude of membrane potential fluctuations can control network frequency even when average firing rates are unchanged. This demonstrates another novel method by which the frequency of network oscillations may be scaled. Because membrane noise introduced by the variability in synaptic inputs could be straightforwardly controlled by the pattern of ongoing activity in a network, this provides a simple, and biophysically-plausible manner in which network frequency could be controlled independently of the firing rate and conductance level of neurons in an intact network.

### Network stability is determined by the balance of excitation and inhibition in magnitude and in time

Some combinations of parameters were found to be unstable, producing network-level oscillations mixed with hypersynchronous burst firing across the entirety of both simulated populations (Fig. 8A). We found that unbalancing the excitation and inhibition received by model RSP neurons either in magnitude or in time was sufficient to induce a transition into this unstable state. This point is illustrated in Fig. 8B, which shows, for example, that increasing the magnitude of recurrent excitation in the model or increasing the inhibitory synapse latency, or a combination of these two factors, generated an unstable condition.

In our model, recurrent excitation represents a positive feedback mechanism for the level of network activity. Similarly, activity-dependent inhibition originating from the FS interneuron population provides negative feedback in proportion to pyramidal cell activity. For this reason, it is unsurprising that an imbalance in these mechanisms would result in an unstable system. However, this observation might provide some insight into the utility of the fast-spiking phenotype, characteristic of FS interneurons. If neurons with a regular-spiking electrophysiological phenotype were tasked with providing feedback inhibition, it is possible that such a network would be unable to operate in a stable manner.

Indeed when spike latencies were measured following an excitatory conductance-based synaptic input waveform in biological layer II/III pyramidal cells and fast-spiking interneurons, we found that the biological RSP neurons responded substantially slower than fast-spiking interneurons (Fig. 8C). Our modeling results (Fig. 8B) suggest that this difference in response times to excitatory conductance inputs would be sufficient to impact network stability in a substantial manner. Many electrophysiological features of FS interneurons seem to be ‘tuned’ in such a way to make them respond to excitatory inputs with the smallest possible latency. They have small time constants, small spike widths, fast synaptic kinetics, and dendrites that seem to be tuned to favor fast conduction of inputs originating at distal locations [74]–[77]. All of these factors could contribute to the fast onset of feedback inhibition, ensuring that the network is endowed with an effective negative feedback mechanism and that network stability is maintained.

### Network sensitivity is modulated by recurrent excitation

In addition to being a critical determinant of network stability, the balance between excitatory and inhibitory current magnitudes was found to control the sensitivity of the network to changes in input. Sensitivity (gain) in the network was measured by first randomly selecting a subpopulation of model excitatory neurons and varying their level of activation with additional DC bias current (Fig. 9A). Following this manipulation, we quantified the change in the firing rate of cells in this subpopulation as a function of the change in driving current (Fig. 9B *i*). When the subpopulation was small, consisting of 1% of the total number of excitatory cells, the additional excitation in the subpopulation was too weak to affect the activity of other cells in the network. For this reason, the frequency-current relationship in this small subpopulation exactly mirrored the relationship of a cell isolated from the local network (appearing as overlapping lines in Fig. 9B *i*), responding with relatively high gain to changes in input current.

In simulations in which large subpopulations (>20% of all excitatory cells) received additional drive, substantial additional recurrent excitation and feedback inhibition were recruited as activity in the subpopulation increased. When the contribution of excitatory and inhibitory currents were balanced in magnitude (G_EE_ = 62.5 nS/cell), network gain was closer to the gain of isolated neurons and small subpopulations (Fig. 9B *i*). In contrast, in simulations in which recurrent excitation was smaller in magnitude, thereby unbalancing the magnitudes of excitation and inhibition, the presence of inhibitory feedback proportional to firing rate decreased the gain of the subpopulation (darker lines). In this condition (i.e. small recurrent excitation), we observed a marked difference between the gain of the subpopulation and the gain of isolated cells or small subpopulations. The separate dependencies of network gain on subpopulation size and recurrent excitation are summarized in Fig. 9B *ii*. These simulations illustrate that inputs impinging upon small subpopulations are coded with high sensitivity while inputs highly correlated across the network may be coded for less strongly when synaptic excitation and inhibition are unbalanced. The implication of this result is that strong recurrent inhibition may limit the sensitivity with which highly correlated inputs are coded across a population. Excitation recruited in equal proportion to inhibition reverses this effect. However, it remains unclear under exactly what conditions excitation and inhibition are closely balanced in a cortical network and how this balance may be adjusted.

## Discussion

In this study, we have investigated the paradigm in which the gamma rhythm arises when feedback inhibition is recruited by principal cell activity, with spikes in all cells driven by noisy fluctuations or synaptic input. Using electrophysiological measurements to provide biophysical constraints on network parameters, we simulated a mixed network of regular-spiking pyramidal (RSP) neurons and fast-spiking (FS) interneurons. These simulations were additionally constrained by published experimental results indicating that individual neurons fire at low rates and with a high degree of irregularity [78]–[82]. Under these assumptions, we have found that oscillations arising from a feedback mechanism emerge robustly in the beta frequency band (15–35 Hz) and not the gamma band (30–80 Hz) when the parameters of model cells are taken from biological cells in a quiescent brain slice.

In further simulations, we have shown that adding a substantial constant conductance source to either cell type, similar to the synaptic conductance received by cells *in vivo* [46], [69], increases the frequency of network oscillations substantially. When the full network was simulated with all cells in the high-conductance state, emergent oscillations occur at a substantially higher frequency even when we control for variables determining the firing rate of individual cells and the efficacy of individual synapses. In fact, simulating the model network with all cells in the high-conductance state produced coherent population-level oscillations ranging from 20–60 Hz depending on the particular choices of DC driving currents, noise, and synapse size. Therefore it seems that under very general conditions, gamma-frequency population oscillations are enabled when constituent neurons are in a high-conductance state, relative to the quiescent state we measure *in vitro*.

The point that small effective membrane time constants promote gamma has been suggested before [70], and some existing models of network gamma include membrane time constants much lower than we measure in the slice [e.g. 9], [26], [72]. The current study makes three novel contributions to this discussion. First, we explicitly measure membrane responsiveness in the brain slice, and show in particular that resting values of membrane conductance in pyramidal cells are too low to support gamma oscillations. Second, we demonstrate that high-conductance membranes in both excitatory and inhibitory neurons contribute to stable oscillations at gamma frequencies, for dramatically different reasons in the two cell types. Third, we show that the inhibitory synaptic conductances responsible for hyperpolarizing principal neurons in a phasic, cycle-to-cycle manner are insufficient to lower membrane time constants in a manner that produces higher-frequency gamma-frequency oscillations (see Fig. 5D), as proposed elsewhere [70], [72]. The apparent discrepancy between these studies and our own could lie in the fact that those studies focused on systems of coupled oscillators. In stark contrast, no cell in our network fires periodically in the absence of input; all activity in the network is generated by fluctuating membrane conductances in the pyramidal neuron population.

At a mechanistic level, we found that the salient effect of altering the time scale of the neuronal membrane was different in the excitatory and inhibitory cells in the network. Specifically, we found the relevant consequence of increasing the conductance of the RSP neuron membrane to be a reduction in the time that the neuronal membrane remained less excitable following coherent inhibitory synaptic input. In contrast to the role of conductance in principal neurons, simulations indicate that background synaptic conductance impinging upon interneurons impacts network frequency in a fundamentally different manner. The decrease in time constant of the interneurons providing feedback inhibition resulted in a decreased latency to spiking following the strong excitatory synaptic inputs which have been shown to impinge upon inhibitory neurons [31], [83]. Decreasing spike latency improves the temporal precision of the circuit's negative feedback mechanism, thereby allowing inhibitory currents to balance excitatory currents in a more effective manner. Under this condition of improved temporal balance, inhibitory currents maintained principal neurons in an inhibited state for a shorter period of time due to a smaller magnitude hyperpolarization, resulting in increased network frequency.

It may seem counterintuitive that the time scale of the principal cell membrane, on the order of tens of milliseconds, would impact the frequency of network oscillations when individual cells fire action potentials with inter-spike intervals of 100 ms or more. However, it is not the time between action potentials that is the relevant quantity in this scenario, but the time between the temporal windows in which the cell is most excitable. Due to the divergence in interneuron-to-pyramidal neuron connections in the network, inhibition is received approximately synchronously by all principal cells in the network every cycle of the oscillation, regardless of whether or not individual neurons have fired an action potential recently or not. Principal neurons are most excitable when inhibition and the associated membrane hyperpolarization have maximally decayed, and because the time constant of the membrane controls this process, it also controls the periods between membrane excitability, and hence, the period of network oscillations.

A key aspect of the analysis contained herein is our stated assumption that constituent neurons, and in particular excitatory neurons, fire sparsely (i.e on a small fraction of the total cycles), during the ongoing oscillation. In the converse case, when excitatory neurons fire at high rates, the conclusions derived from our analysis of recovery times after inhibition do not hold generally. In the sparse-firing scenario, in which spikes are initiated following noise-induced threshold crossings, the transmembrane voltage of principal cells recovers to a stable, subthreshold voltage following inhibitory input. The time constant of the principal cell membrane has a substantial effect on this recovery time after inhibition. In contrast, the recovery time of a tonically-spiking neuron following an inhibitory input is less sensitive to membrane time constant. Instead, an inhibitory input will perturb the timing of the next spike in a manner described by its phase-resetting curve [15], [84], [85], which is unlikely to be altered qualitatively by the presence of added conductance, provided one controls for changes in firing rate. Interestingly, this discrepancy implies that reduced gamma network models, in which single oscillating neurons are used to represent the summed activity of a coherent population [e.g. 21], are unlikely to depend on membrane conductance or noise magnitude in the same manner. Although the simulated interneurons in this study fire at higher average rates than the excitatory cells, they are still excited by noise and synaptic input rather than intrinsic drive, implying that phase-response analyses and highly reduced models of the GABAergic population are also likely to lead to different results than those we observe.

Our approach in this study has largely been an experimentally-anchored variety of the analytical approach previously developed by Brunel and colleagues [35], [36], [86]. Although the work of Brunel and colleagues shares points of interest with the current one, there are important differences between our approaches and findings as well. In order to make the problem analytically tractable, Geisler et al. focused on the effects of the ratio between excitatory and inhibitory currents in the network while imposing the condition that this ratio be equal in both excitatory and inhibitory neurons. Increasing this ratio was found to either increase or decrease network frequency, depending on the relative phases of the two components. They further showed that factors including the effective membrane time constant can change the effect of the drive ratio by changing phasing, but did not report the effects of membrane time constant on network frequency. We constrain our study to empirically measured phase relationships and, in accordance with measured data [26], [31], break the constraint of equal ratios in the two classes of postsynaptic neurons. Consistent with past results [26], [36], we find that even temporary increases in the relative level of excitatory drive gave rise to lower network frequencies in the subsequent cycles of the population rhythm. Regardless of the approach, our results are in general agreement with those from Geisler et al. that intrinsic properties of constituent neurons may impact network-level gamma-frequency oscillations, in contrast with previous suggestions that the only relevant time scales determining network frequency relate to the kinetics of synaptic inhibition.

An advantage of studying network activity in a spiking model is the capability to investigate the fine structure of events, such as the hypersynchronous bursting observed in the model network. During epochs of excessive excitation, the model interneuron population spikes coherently in the 150–250 Hz frequency band before individual cells enter a state of depolarization block (Fig. 8A *ii*). Similar population-wide activation is observed in the neocortex of some patients with epilepsy during interictal spikes in the EEG waveform. In these recordings, measured local field potentials display an increase in energy at high frequencies (>150 Hz) (B. Greger, personal communication). Interestingly, as in this model, depolarization block of local neurons has been proposed as a generative mechanism for this observed field potential waveform (B. Greger, personal communication). Additionally, activation of spike-frequency adaptation mechanisms in model neurons following hypersynchronous activation produces a period of relative network quiescence commonly lasting several hundred milliseconds, another feature observed experimentally (B. Greger, personal communication). As an imbalance in excitatory and inhibitory elements has been proposed as an underlying cause of many forms of epilepsy, it is possible that the instability we observe in the model could be related to the pathological activation underlying interictal bursting in epileptic individuals.

Balanced excitatory-inhibitory networks may arise, generally, as a result of two disparate mechanisms. This balance may occur when an external excitatory input source drives proportional feedforward inhibition, a phenomenon thought to be necessary for the temporal gating of feedforward inputs [87], [88]. Alternatively, balanced excitation and inhibition may occur in feedback networks in which principal cell activity recruits proportional feedback inhibition [26], [36], [86], [89] or in networks in which a balance is achieved through a combination of feedforward and feedback inhibition [90]. If this balance is achieved in a feedback manner and if the delay associated with feedback inhibition is sufficiently large, the activity of such a network will be prone to periodic oscillation [62], [86], [91]. This follows generally from control theoretical results; systems including delayed negative feedback oscillate under broad conditions, particularly when the system involves substantial positive feedback as well [92].

The interpretation that increased conductance in the fast-spiking interneuron population leads to faster network oscillations by decreasing spike latency is consistent with these ideas. Control theory states that reducing the inherent delay of the negative feedback process will lead to a system with smaller amplitude, faster oscillations, as is illustrated in Fig. 6. If the excitatory-inhibitory delay were decreased further, we would predict that the network described in this study would approach the asynchronous, balanced state described elsewhere [86], [89]. Control theory also states that a decrease in the delay associated with negative feedback will generally improve the stability of a system containing both positive and negative feedback mechanisms. We found the increase in interneuron membrane response speed associated with added background synaptic conductance sufficient to impact the stability of our modeled network by a substantial amount. In fact, given the broad range of membrane time constants described across neuronal cell types, our results indicate that feedback inhibition mediated by non-fast-spiking neurons would be unlikely to maintain the stability of a network with any appreciable recurrent excitatory connectivity. Interestingly, the fact that control theoretical results from simple systems involving positive and negative feedback elements are consistent with results from our simulations and with experimental findings on the gamma rhythm bolsters the argument that, in some conditions, population oscillations may arise in the brain as a result of recurrent excitation and the commensurate feedback inhibition necessary to maintain stability [26], [35], [36], [62].

## Supporting Information

Figure S1 The dependencies of network frequency on conductance state of RSP neurons and FS neurons are qualitatively similar in the presence of electrical synapses and faster FS-FS inhibition. **A** Power spectra of field potential approximation from simulations in which all constituent neurons are in a low-conductance state (black curve) and high-conductance state (red), when only RSP neurons are in a high-conductance state (blue), and when only FS interneurons are in the high conductance state (green). Results are qualitatively similar to that shown in Fig. 4C. **B** Distributions of firing rates RSP neurons (*i*) and FS interneurons (*ii*) in the simulations described in *A*. Compare with Fig. 4B. **C** Average RSP neuron membrane potential derivative during recovery phase (*i*) and excitation-inhibition delay (*ii*) for the four cases described in *A*. Compare with Figs. 5C and 6A. **D** Cycle-averaged RSP neuron membrane potential for the four cases described in *A*. Panels *C–D* illustrate that the same mechanisms controlling frequency in Figs. 4–6 (main text) are responsible for the change in frequency described in *A*.
(TIF)

Table S1 Maximal synaptic conductance values used for Supporting Fig. S1.
(DOC)

Text S1 Detailed description of the simulations depicted in Fig. S1.
(DOC)
