# Supplementary figures and images for: Membrane Properties and the Balance between Excitation and Inhibition Control Gamma-Frequency Oscillations Arising from Feedback Inhibition
Source: PLoS Comput Biol. 2012 Jan 19;8(1):e1002354. doi: 10.1371/journal.pcbi.1002354 (PMC3261914; doi:10.1371/journal.pcbi.1002354)

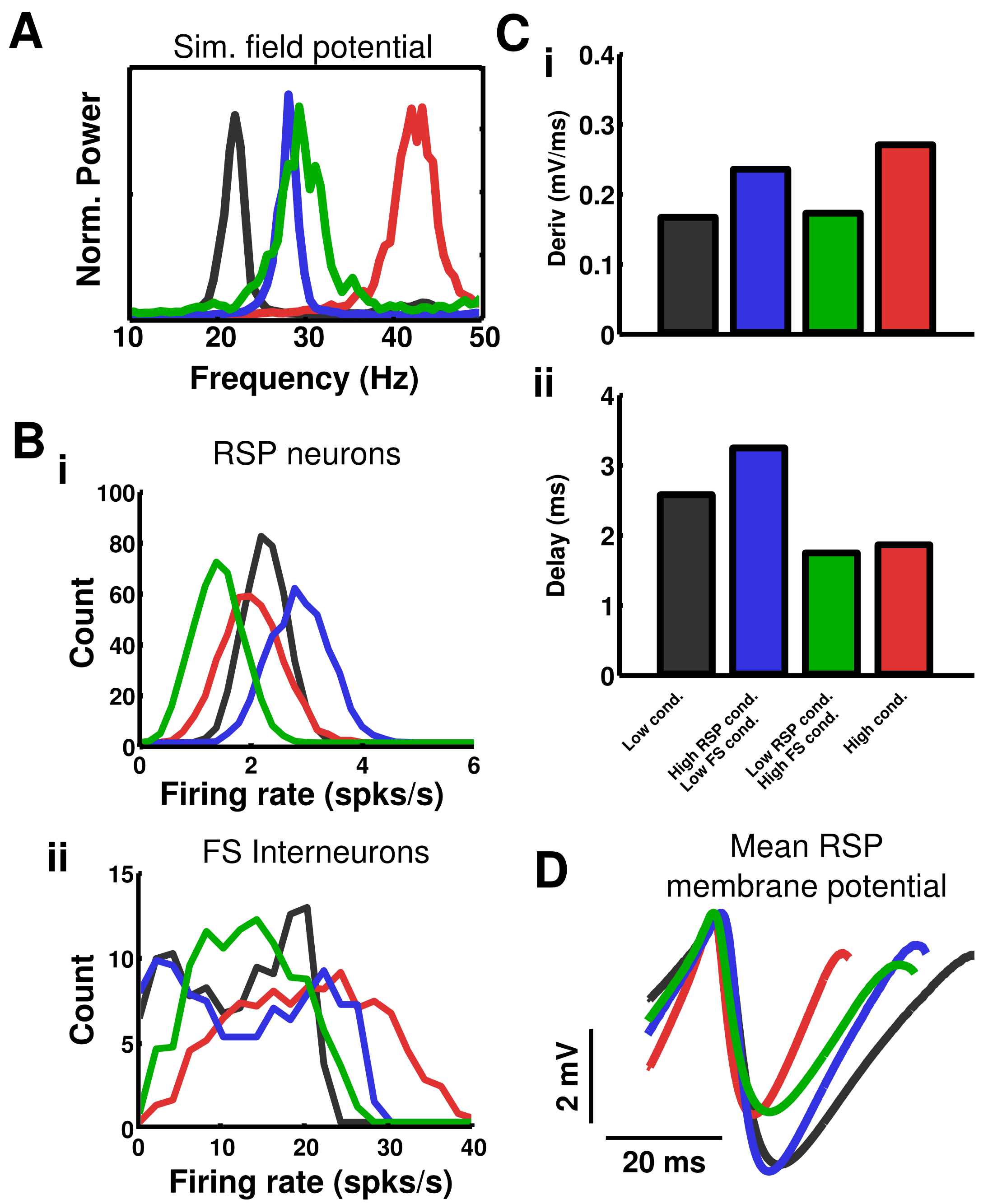

Supplement: Figure S1 — The dependencies of network frequency on conductance state of RSP neurons and FS neurons are qualitatively similar in the presence of electrical synapses and faster FS-FS inhibition. A Power spectra of field potential approximation from simulations in which all constituent neurons are in a low-conductance state (black curve) and high-conductance state (red), when only RSP neurons are in a high-conductance state (blue), and when only FS interneurons are in the high conductance state (green). Results are qualitatively similar to that shown in Fig. 4C. B Distributions of firing rates RSP neurons (i) and FS interneurons (ii) in the simulations described in A. Compare with Fig. 4B. C Average RSP neuron membrane potential derivative during recovery phase (i) and excitation-inhibition delay (ii) for the four cases described in A. Compare with Figs. 5C and 6A. D Cycle-averaged RSP neuron membrane potential for the four cases described in A. Panels C–D illustrate that the same mechanisms controlling frequency in Figs. 4– 6 (main text) are responsible for the change in frequency described in A. (TIF) [file pcbi.1002354.s001.tif]
