# Supplementary material for: Membrane Properties and the Balance between Excitation and Inhibition Control Gamma-Frequency Oscillations Arising from Feedback Inhibition
Source: PLoS Comput Biol. 2012 Jan 19;8(1):e1002354. doi: 10.1371/journal.pcbi.1002354 (PMC3261914; doi:10.1371/journal.pcbi.1002354)
Supplement: Table S1 — Maximal synaptic conductance values used for Supporting Fig. S1. (DOC) [file pcbi.1002354.s002.doc]

**Table S1**

| **Low conductance synapses** | **Value** | **Units** |
| --- | --- | --- |
| $\bar{G}_{EE}$ | 45 | nS |
| $\bar{G}_{EI}$ | 140 | nS |
| $\bar{G}_{IE}$ | 210 | nS |
| $\bar{G}_{II}$ | 125 | nS |
|  |  |  |
| **High conductance synapses** |  |  |
| $\bar{G}_{EE}$ | 100 | nS |
| $\bar{G}_{EI}$ | 600 | nS |
| $\bar{G}_{IE}$ | 350 | nS |
| $\bar{G}_{II}$ | 250 | nS |
