# Supplementary material for: Membrane Properties and the Balance between Excitation and Inhibition Control Gamma-Frequency Oscillations Arising from Feedback Inhibition
Source: PLoS Comput Biol. 2012 Jan 19;8(1):e1002354. doi: 10.1371/journal.pcbi.1002354 (PMC3261914; doi:10.1371/journal.pcbi.1002354)
Supplement: Text S1 — Detailed description of the simulations depicted in Fig. S1. (DOC) [file pcbi.1002354.s003.doc]

**Supporting Text S1**

The simulations illustrated in Supporting Fig. S1 were as described in *Materials and Methods* with two modifications to the nature of synaptic transmission within the population of FS interneurons. First, electrical synapses connecting only FS interneurons were added. Second, the decay time constant of FS-FS inhibition was shortened to illustrate the robustness of results with respect to this parameter. Here we describe in detail these modifications.

Gap junctions: Each pair of interneurons was bi-directionally coupled with a probability p = 0.3. Although connection probabilities for FS interneurons in neocortex have found to be somewhat higher than this value for adjacent neurons, this value approximates the probability of connection between all cells in a small ‘neighborhood’ and yields an average value of 30 electrical synapses per cell, consistent with published reports [1]. Individual gap junction conductances were uniformly distributed between 0.225 nS and 0.375 nS yielding “coupling coefficients” of between 3.2% and 5.2% for each pair of FS interneurons in the quiescent condition, as observed experimentally in superficial somatosensory cortex [2]. Synapse magnitudes are described in Supporting Table S1.

Inhibitory synapse kinetics: The decay time constant of ionotropic chemical synaptic transmission between pairs of interneurons was decreased from 5.0 ms to 2.6 ms, as reported elsewhere [3].

**References**

1. Connors BW, Long MA (2004) Electrical synapses in the mammalian brain. Annu. Rev. Neurosci. 27: 393-418. doi:10.1146/annurev.neuro.26.041002.131128

2. Tamás G, Buhl EH, Lörincz A, Somogyi P (2000) Proximally targeted GABAergic synapses and gap junctions synchronize cortical interneurons. Nat. Neurosci. 3: 366-371. doi:10.1038/73936

3. Galarreta M, Hestrin S (2002) Electrical and chemical synapses among parvalbumin fast-spiking GABAergic interneurons in adult mouse neocortex. Proc. Natl. Acad. Sci. U.S.A 99: 12438-12443. doi:10.1073/pnas.192159599
